# Electrical data of 10W, 40W, 80W, and 250W photovoltaic modules under the aging condition: Tested by a Solar Manufacturer Company in Bangladesh

**DOI:** 10.1016/j.dib.2023.108989

**Published:** 2023-02-15

**Authors:** Ahmed Al Mansur, Md. Ruhul Amin, Md. Imamul Islam, ASM Shihavuddin

**Affiliations:** a Department of Electrical and Electronic Engineering, Green University of Bangladesh, Dhaka, Bangladesh; b Department of Electrical and Electronic Engineering, Islamic University of Technology, Gazipur, Dhaka, Bangladesh

**Keywords:** Nonuniformly aged module, Photovoltaic degradation, PV characteristics, Tested data, Commercial sun simulator

## Abstract

The health monitoring system of photovoltaic modules throughout their lifespan is an important research topic. The dataset of aged PV modules is required to investigate the performance of the aged PV array for simulation work. Different aging factors are responsible for decreasing the output power of aged PV modules and increasing the degradation rate. In addition, mismatch power losses are increases with the nonuniformity of aged PV modules due to different aging factors. In this work, four datasets of 10W, 40W, 80W, and 250W PV modules are collected under nonuniform aging conditions. Each dataset contains forty modules with a four-year aged average. The average deviation of each electrical parameter of the PV modules can be calculated from this data. Moreover, a correlation can be developed between the average deviation of electrical parameters and mismatch power loss in PV array modules under early aging conditions.

**Specifications Table**

**Table:** 

Subject	Renewable Energy, Sustainability, and the Environment
Specific subject area	Testing of 10W, 40W, 80W, and 250W power-ratted photovoltaic modules under aging conditions using a commercial sun simulator in an indoor test environment.
Type of data	Table Figure
How the data were acquired	Four datasets of 10W, 40W, 80W, and 250W PV modules were acquired and each dataset contains forty modules. The data were acquired by testing each module utilizing a commercial sun simulator (IV Tester) in indoor conditions. The aged PV modules are collected from four different off-grid PV plants where the modules were exposed to the outdoor environment for four years on average. The aging period is considered from the date of installation of new PV modules for each array. Testing Instruments: i) Commercial IV Tester: Size: 2400mm × 1650mm × 900mm; Weight: 600kg; Type: Light shoots vertically from the button up; Test Mode: Single time flash test; Light Source: Long arc pulsed Xenon lamp in accord with IEC60904-9 spectral irradiance distribution requirements; Measurement Error: ≤±0.5%; Irradiation No Uniformity: ≤±2% A-Class. ii) Software: Microsoft Office for data processing from the commercial sun simulator storage system.
Data format	Raw
Description of data collection	With the assistance of the Electro Group, Dhaka, Bangladesh these data were gathered empirically utilizing a commercial technology under standard test conditions. Electro Group is a solar panel manufacturer company. Which provides the electrical parameters such as maximum power, voltage, current, open circuit voltage, and current for each tested PV module.
Data source location	*Institution: Electro Group* *City: Dhaka* *Country: Bangladesh* *Latitude and longitude: 23.90206486536505, 90.30576583735689*
Data accessibility	Repository name: Harvard Dataverse Data identification number: https://doi.org/10.7910/DVN/MIONZF Direct URL to data: https://dataverse.harvard.edu/dataset.xhtml?persistentId=doi:10.7910/DVN/MIONZF
Related research article	Al Mansur, A., Amin, M.R., ul Haq, M.A., Maruf, M.H., Mottalib, M.M., Ashique, R.H. and Shihavuddin, A.S.M., 2022. Mitigation of mismatch power loss in aged photovoltaic arrays following a comparative investigation into module rearrangement techniques. Energy Reports, 8, pp.1896-1906. https://doi.org/10.1016/j.egyr.2022.01.013[1].

## Value of the Data

• These datasets are useful to investigate aged PV power plant-based research.
• Academic and industrial researchers will be benefited from these data.
• The data are used to investigate the mismatch power losses of PV power plants through simulation work with experimental validation.
• Further experimentation and comparative analysis can be done to investigate the PV power degradation rate considering the electrical parameters of the tested datasets.

## Objective

In photovoltaic research, mismatch power loss minimization is a hot topic. This power loss increases significantly due to the nonuniform aging of PV modules in an array. Four datasets of ununiformly aged PV modules (10W, 40W, 80W, and 250W) are provided in this work. These datasets are very useful to investigate the mismatch power losses of four different power-ratted photovoltaic arrays. Each dataset consists of 40 modules. Therefore, six array configurations, 2 × 20, 4 × 10, 5 × 8, 2 × 2, 10 × 4, and 8 × 5 can be used to investigate the relation between the array size and mismatch power losses. Moreover, using these datasets different module rearrangement techniques can be investigated by simulation considering power losses, array dimensions, array output power for aged PV arrays [1].

## 1 Data Description

**Fig. 1:**
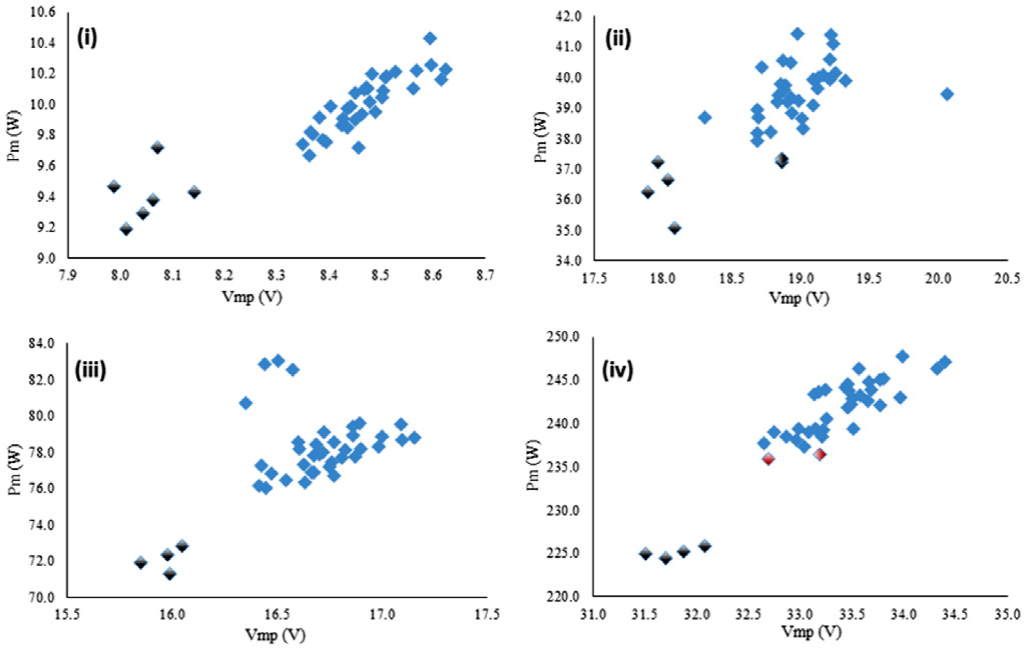
Correlation between Vmp and Pm of aged PV modules (i) 10W, (ii) 40W, (iii) 80W, and (iv) 250W.

In this work, four datasets of the electrical parameters of four years aged PV modules with various ratings are provided. The datasets are available in the HARVARD Dataverse website and the filename is “Tested Dataset of 10W, 40W, 80W and 250W Photovoltaic Modules under Aging Condition” [2]. Each dataset contains 40 modules with ratings of 10W, 40W, 80W, and 250W. Tables 1 represent the average value of the electrical parameters of 10W, 40W, 80W, and 250W PV modules. Where the data shows that the current and voltage rating increased with the power rating increment. The voltage (Voc) is almost the same for 40W and 80W modules however the current (Isc) is doubled to maintain the power rating. In 250W modules, both the voltage and current rating are increased with the power. The percentage of fill factors of 10W, 40W, 80W, and 250W PV modules are 73.13, 76.35, 74.51, and 78.15 respectively. Where the minimum fill factor (73.13) shows for lower power rated, 10W modules and the maximum (78.15) for the higher power-rated 250W modules. Fig. 1 illustrates the correlation between each tested PV module's maximum power point voltage (Vmp) and the maximum power (Pm). The gradient color indicates the degraded modules from the average power points. The degradation of a PV module starts with discoloration and delamination at the early aging conditions which creates nonuniformity among the cells of a PV module considering current generation. The degraded PV modules causes the nonuniformity among the PV array considering the current and power generation. Therefore the percentage of nonuniformity of an aged module in an array can be calculated from the deviation of its output power from the average value. These degraded modules are the key source of the mismatch power losses in the large PV arrays [1].

**Table 1:** Electrical parameters of the aged PV modules dataset.

	Electrical parameters of the aged PV modules (average)					
Module rating (W)	Open Circuit Voltage, Voc (V)	Short Circuit Current, Isc (A)	Maximum Power Voltage, Vmp (V)	Maximum Power Current, Imp (A)	Maximum Power, Pm (W)	Fill Factor (%)
10	10.39	1.30	8.40	1.17	9.91	73.13
40	22.98	2.22	18.87	2.07	39.12	76.35
80	21.01	4.96	16.64	4.67	77.72	74.51
250	40.40	7.60	33.23	7.22	240.14	78.15

**Fig. 2:**
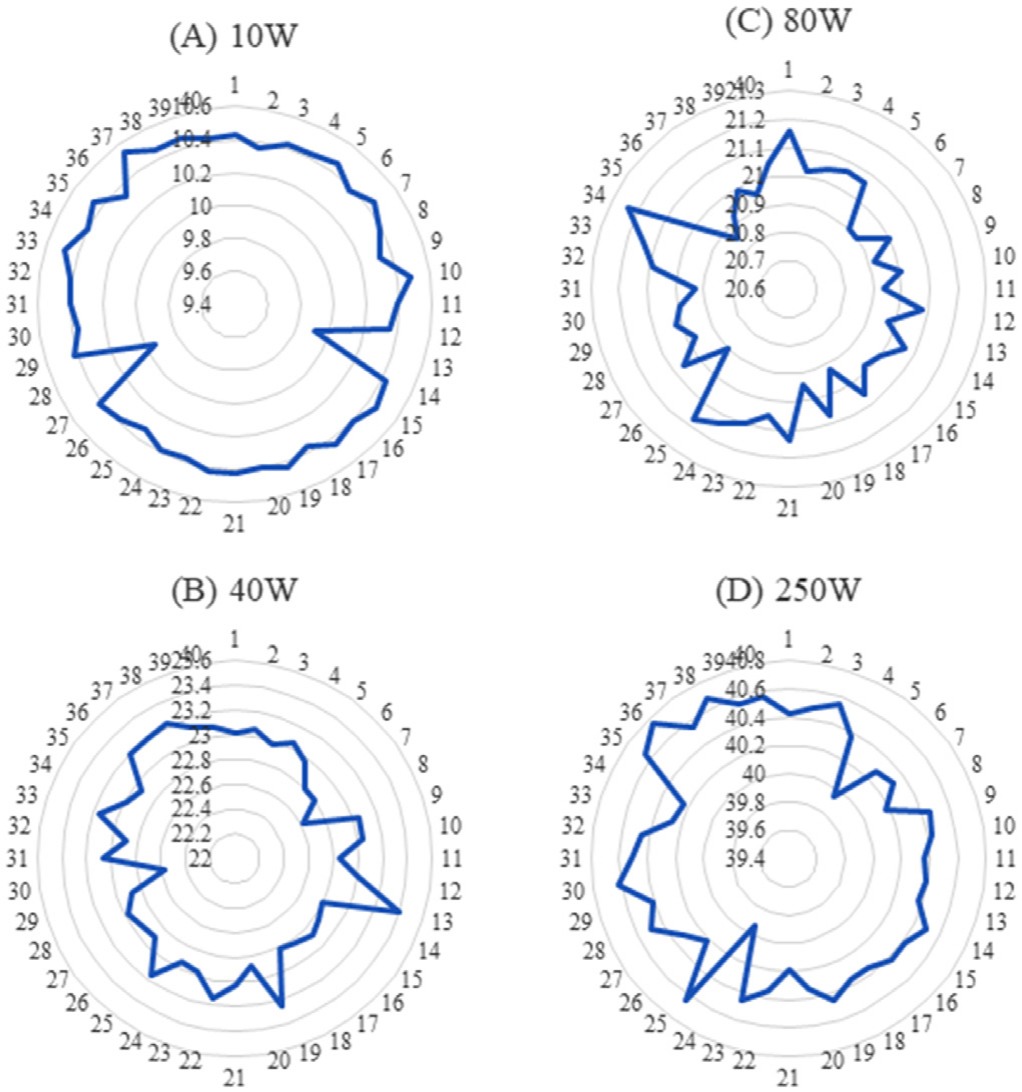
Open circuit voltage of aged PV modules (A) 10W, (B) 40W, (C) 80W, and (D) 250W.

**Fig. 3:**
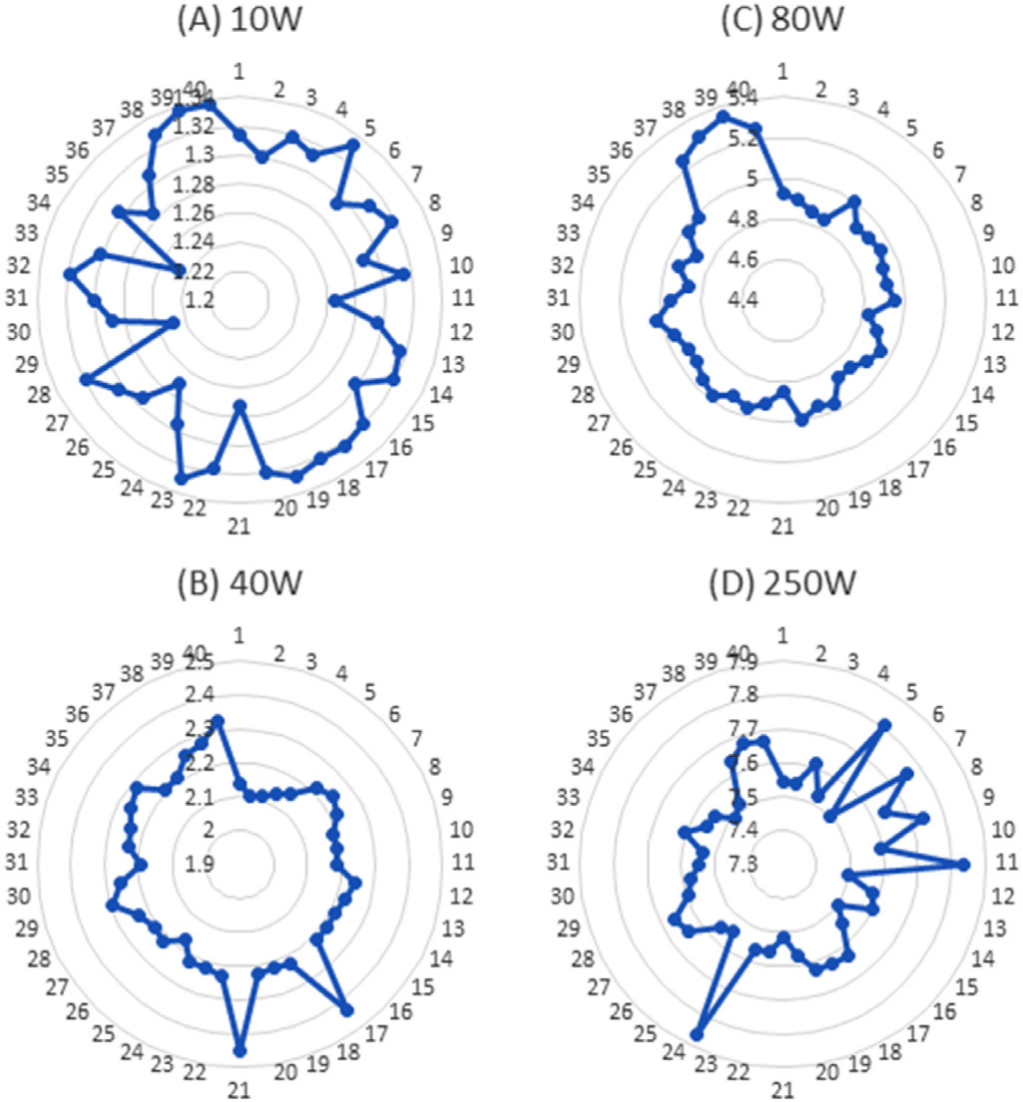
Short circuit of aged PV modules (A) 10W, (B) 40W, (C) 80W, and (D) 250W.

**Fig. 4:**
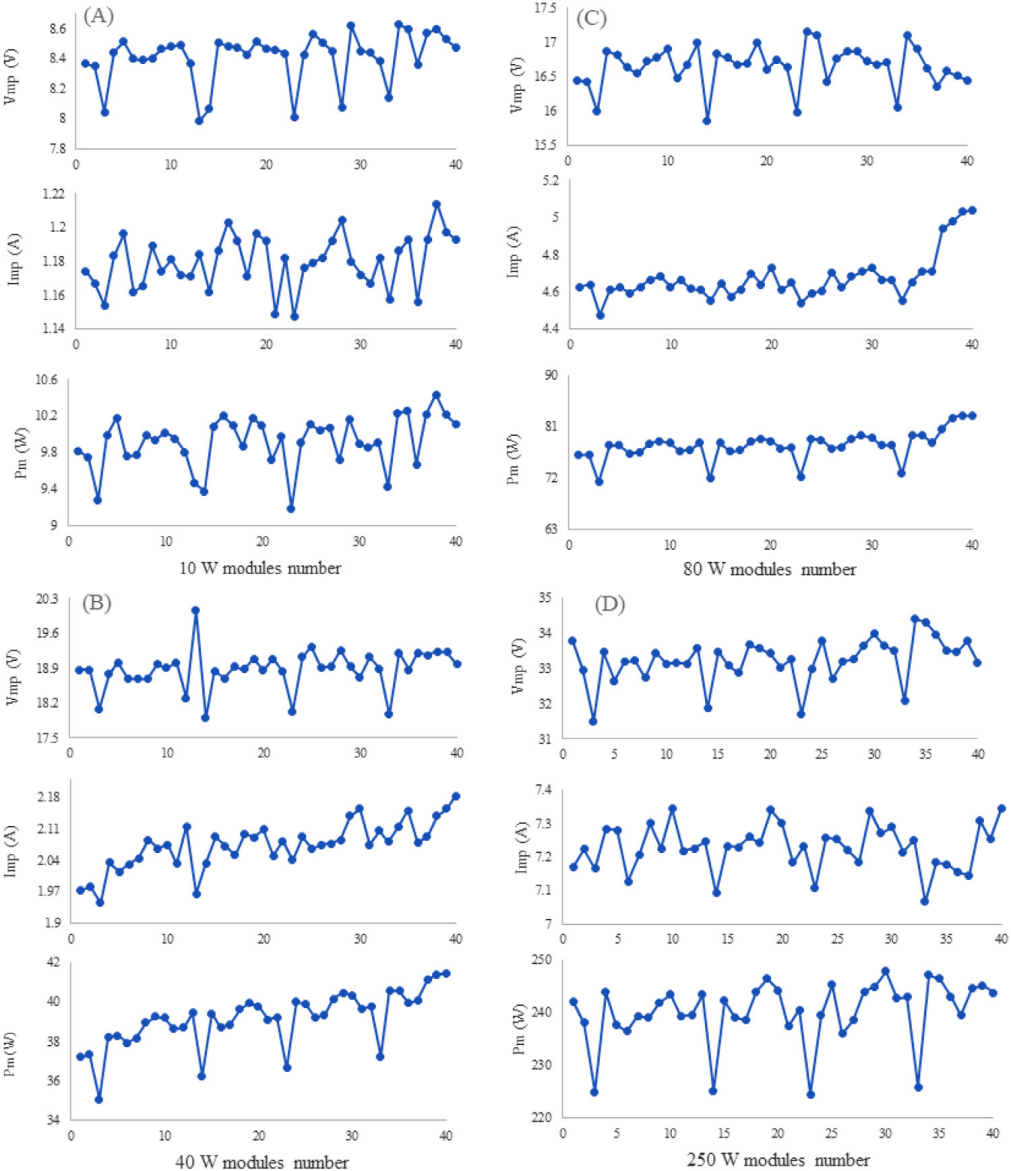
Maximum power point voltage (Vmp), current (Imp), and power (Pm) of the aged PV modules dataset (A) 10W, (B) 40W, (C) 80W, and (D) 250W.

Fig. 2 shows the deviation of the open circuit voltage (Vco) of the 10W, 40W, 80W, and 250W PV modules. Where the most deviation is found in 80W PV modules due to aging factors and therefore the average fill factor is lower than the 40W and 250W modules. Fig. 3 illustrates the short circuit current (Isc) of the 10W, 40W, 80W, and 250W PV modules. The relationship between the module parameters (Voc and Isc) and mismatch power losses is not investigated in the related research paper [1]. Therefore, further research can be done using these datasets. Fig. 4 shows the Maximum power point voltage (Vmp), current (Imp), and power (Pm) of the aged PV modules of 10W, 40W, 80W, and 250W ratings. Where a correlation between the Imp and Pm is already identified in the published research article [1]. However, the relation between the Vmp, Voc with the Pm, and mismatch power losses are not investigated yet.

## 2 Experimental Design, Materials and Methods

**Fig. 5:**
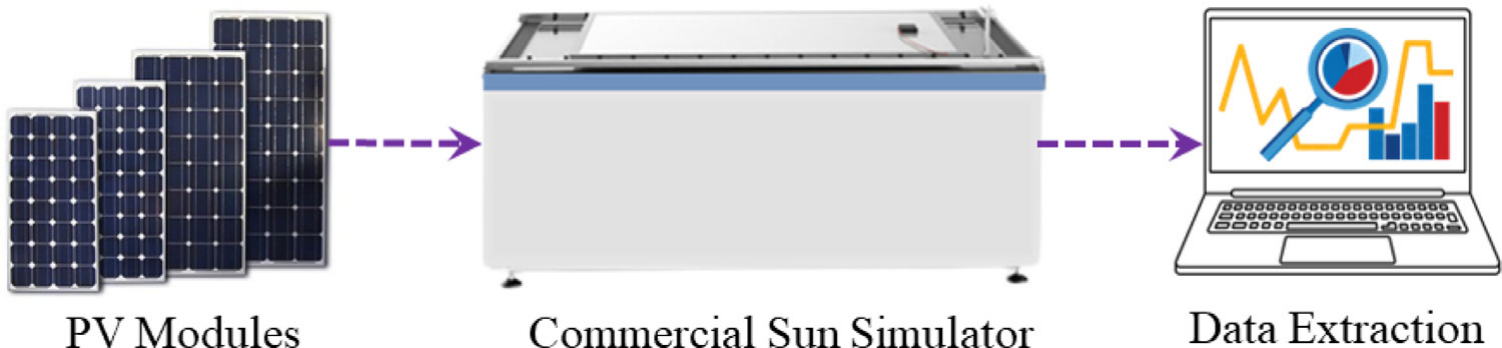
The schematic diagram of the PV module testing system using a commercial IV tester.

The aged photovoltaic modules are collected from four different power-rated PV plants such as 400W, 1600W, 3200W, and 10000W. 10W and 40W modules are collected from 400W and 1600W PV plants respectively. 80W and 250W modules are collected from 3200W and 10000W PV power plants respectively [1]. These four sets of different power-rated PV modules are tested in a PV manufacturer company, Electro Group, Dhaka, Bangladesh. Utilizing a commercial sun simulator [3], each panel was examined under Stander Test Condition (STC) [4]. Fig. 5 illustrates the experimental procedure of collecting data by employing the commercial platform. A xenon light-based sun simulator was used to provide artificial sunlight for the PV modules and the output electrical parameters were measured by an IV tracer and stored in the PC. Specific software is used for data extraction which has been provided by the manufacturer company of the commercial IV tracer. The extracted data can be accessed by Microsoft excel.

## Ethics Statements

The authors, confirm that the dataset of solar modules was tested practically form a renowned solar manufacturer company, ESL in Bangladesh. The dataset was verified and reviewed and the related research paper [1] is published by the same authors. The authors have read and followed the ethical requirements for publication in Data in Brief.

## CRediT authorship contribution statement

**Ahmed Al Mansur:** Conceptualization, Methodology, Writing – original draft, Data curation, Validation, Formal analysis, Investigation. **Md. Ruhul Amin:** Supervision, Conceptualization, Data curation, Writing – review & editing. **Md. Imamul Islam:** Conceptualization, Writing – original draft, Software, Formal analysis, Data curation. **ASM Shihavuddin:** Supervision, Project administration, Investigation, Data curation.

## Declaration of Competing Interest

The authors declare that they have no known competing financial interests or personal relationships that could have appeared to influence the work reported in this paper.

## Data Availability

Tested Dataset of 10W, 40W, 80W and 250W Photovoltaic Modules under Aging Condition (Original data) (Dataverse).
